# Supplementary material for: Ascent rate and the Lake Louise scoring system: An analysis of one year of emergency ward entries for high-altitude sickness at the Mustang district hospital, Nepal
Source: PLoS One. 2022 Oct 27;17(10):e0276901. doi: 10.1371/journal.pone.0276901 (PMC9612449; doi:10.1371/journal.pone.0276901)
Supplement: S1 Table — (PDF) [file pone.0276901.s001.pdf]

S1 Table: Socio-demographic characteristics, comorbidities and AMS severity distribution

Category	Variables	Frequency (Percent of Patient [95% CI])	AMS severity			P-value[b]
			No	Mild	Moderate & Severe	
**Gender**	Female	47 (44.8% [35.3 - 54.3])	12 (25.5%)	17 (36.2%)	18 (38.3%)	0.105
	Male	58 (55.2% [45.7 - 64.7])	15 (25.9%)	31 (53.4%)	12 (20.7%)	
**Age**	16-30	30 (28.6% [20.0 - 37.2])	7 (23.3%)	18 (60%)	5 (16.7%)	0.038
	31-60	46 (43.8% [34.3 – 53.3])	10 (21.7%)	16 (34.8%)	20 (43.8%)	
	≥ 61	29 (27.6% [19.0 - 36.2])	10 (34.5%)	14 (48.3%)	5 (17.2%)	
**Nationality**	Nepali	78 (74.3% [65.9 - 82.7])	24 (30.8%)	32 (41%)	22 (28.2)	0.106
	Non-Nepali	27 (25.7% [17.3 - 34.1])	3 (11.1%)	16 (59.3%)	8 (29.6%)	
**Smoking history**	Yes	17 (16.2% [9.2 - 23.2])	9 (52.9%)	6 (35.3%)	2 (11.8%)	0.015
	No	88 (83.8% [76.8 – 90.8])	18 (20.8%)	42 (47.7%)	28 (31.8%)	
**Alcohol intake**	Yes	27 (25.7% [17.3 - 34.1])	2 (7.4%)	22 (81.5%)	3 (11.1%)	0.001
	No	78 (74.3% [65.9 – 82.7])	25 (32.1%)	26 (33.3%)	27 (34.6%)	
**Comorbid illness**	Yes	29 (27.6% [19.0 - 36.2])	10 (34.5%)	11 (37.9%)	8 (27.6%)	0.418
	No	76 (72.4% [63.8 - 81])	17 (22.4%)	37 (48.7%)	22 (28.9%)	
**Medication history**	Yes	20 (19.0% [11.5 - 26.5])	3 (15%)	9 (45%)	8 (40%)	0.327
	No	85 (81% [73.5 – 88.5])	24 (28.2%)	39 (45.9%)	22 (25.9%)	

a Percentage was calculated for the frequency of the category variable.
b The chi-square test was used for AMS observed with in each group in the study population.
