# Supplementary material for: Ascent rate and the Lake Louise scoring system: An analysis of one year of emergency ward entries for high-altitude sickness at the Mustang district hospital, Nepal
Source: PLoS One. 2022 Oct 27;17(10):e0276901. doi: 10.1371/journal.pone.0276901 (PMC9612449; doi:10.1371/journal.pone.0276901)
Supplement: S2 Table — (PDF) [file pone.0276901.s002.pdf]

S2 Table: Distribution of symptoms' intensity and symptoms' presence in patients with AMS

		Distribution of symptoms' Intensity (Percent of Patient [95% CI])			
		Fatigue	Headache	Dizziness	Gastrointestinal
**Symptom Intensity**	No	4 (3.8% [0.1 - 7.5])	25 (23.8% [15.7 - 32])	29 (27.6% [19.1 - 36.2])	36 (34.3% [25.2 - 43.4])
	Mild	57 (54.3% [44.8 - 63.8])	42 (40.0% [30.6 - 49.4])	49 (46.7% [37.1 - 56.2])	42 (40.0% [30.6 - 49.4])
	Moderate	42 (40.0% [30.6 - 49.4])	34 (32.4% [23.4 - 41.3])	27 (25.7% [17.4 - 34.1])	22 (21.0% [13.2 - 28.7])
	Severe	2 (1.9% [0 - 3.8])	4 (3.8% [0.1 - 7.5])	0	5 (4.8% [0.7 - 8.8])
**AMS**	No	25 (92.6%)	2 (0.7%)	10 (37%)	12 (44.4%)
	Yes	76 (97.4%)	78 (100%)	66 (84.6%)	57 (73.1%)
