# Supplementary material for: Ascent rate and the Lake Louise scoring system: An analysis of one year of emergency ward entries for high-altitude sickness at the Mustang district hospital, Nepal
Source: PLoS One. 2022 Oct 27;17(10):e0276901. doi: 10.1371/journal.pone.0276901 (PMC9612449; doi:10.1371/journal.pone.0276901)
Supplement: S3 Table — (PDF) [file pone.0276901.s003.pdf]

S3 Table: Variation in vital signs with AMS severity

Classification	No of Cases (%)	Mean Body temperature	Mean Pulse rate	Mean SPO2	Mean Arterial Pressure
**No AMS**	27 (25.7%)	97.1 ± 1.2	95.2 ± 16.3	86.3 ± 8.9	104.2 ± 14.2
**Mild**	48 (45.7%)	97.3 ± 1.4	88.9 ± 22.9	82.5 ± 12.7	100.7 ± 14.6
**Moderate & Severe**	30 (28.6%)	96.8 ± 2.5	94.2 ± 19.5	83.6 ± 14.7	103.4 ± 13.9
