# Supplementary material for: Ascent rate and the Lake Louise scoring system: An analysis of one year of emergency ward entries for high-altitude sickness at the Mustang district hospital, Nepal
Source: PLoS One. 2022 Oct 27;17(10):e0276901. doi: 10.1371/journal.pone.0276901 (PMC9612449; doi:10.1371/journal.pone.0276901)
Supplement: Attachment — Submitted filename: Point-by-point response to reviewers.docx [file pone.0276901.s005.docx]

| **Editor's comments:** | **Response** |
| --- | --- |
| 1. Please ensure that your manuscript meets PLOS ONE's style requirements, including those for file naming. The PLOS ONE style templates can be found at  https://journals.plos.org/plosone/s/file?id=wjVg/PLOSOne_formatting_sample_main_body.pdf and  https://journals.plos.org/plosone/s/file?id=ba62/PLOSOne_formatting_sample_title_authors_affiliations.pdf | We agree and now have updated manuscript following the guideline. |
| 2. Please provide additional details regarding participant consent. In the ethics statement in the Methods and online submission information, please ensure that you have specified (1) whether consent was informed and (2) what type you obtained (for instance, written or verbal, and if verbal, how it was documented and witnessed). If your study included minors, state whether you obtained consent from parents or guardians. If the need for consent was waived by the ethics committee, please include this information.  If you are reporting a retrospective study of medical records or archived samples, please ensure that you have discussed whether all data were fully anonymized before you accessed them and/or whether the IRB or ethics committee waived the requirement for informed consent. If patients provided informed written consent to have data from their medical records used in research, please include this information. | Our study is retrospective study of medical records. Records were anonymized before the researchers had access to them. We have updated ethical considerations in Methods section with clarifying statement. |
| 3. We note that you have stated that you will provide repository information for your data at acceptance. Should your manuscript be accepted for publication, we will hold it until you provide the relevant accession numbers or DOIs necessary to access your data. If you wish to make changes to your Data Availability statement, please describe these changes in your cover letter and we will update your Data Availability statement to reflect the information you provide. | We are providing the github repository link. Once the article has been accepted, we will make the github repository public. |
| 4. Ethics statement appears in the Methods section of the manuscript AND at the end of the manuscript: Your ethics statement should only appear in the Methods section of your manuscript. If your ethics statement is written in any section besides the Methods, please delete it from any other section. | We agree and have removed ethics statement from the end of the manuscript. |
| 5. Please include captions for your Supporting Information files at the end of your manuscript, and update any in-text citations to match accordingly. Please see our Supporting Information guidelines for more information: http://journals.plos.org/plosone/s/supporting-information. | We have now updated the manuscript to include captions of SI tables. |
| Additional Editor Comments:  Please carefully see the comments from the reviewers and revise the manuscript in accordance with the comments. |  |
| **Reviewer 1 comments:** | **Response** |
| Reviewer #1: This is an important study reporting the risk factors of AMS. Please correct the following points. |  |
| 1. You mentioned odds ratio in the Abstract, but not in the Result. It is better to describe odds ratio in the Result. | The odds ratio in the abstract is from the explanation of figure 2, which comes under the result heading. The odds ratio is calculated as ratio of $P\left( 1day \vert X \right)/P\left( 3day \vert X \right)$, where P(.\|X) is conditional probability of developing moderate or severe AMS. The reason we have not further discussed is because distribution o[f underlying probability is shown in the figure (it is counterfactual probability plot) and we have explained textually about odds-ratio in the figure explanation. |
| 2. In the Methods, you did not mention the detail about the Bayesian-ordered logistic model. Please describe more about the study design or analysis. The sentence “The responsive variable, AMS severity, is an ordered variable with three levels…” in 3.3 is usually mentioned in the Methods. | (a) In statistical analysis, we wrote bayesian multivariate logistic regression, which we now have changed to Bayesian ordered logistic regression. We did not use informed priors, which would have brought out the strength of the Bayesian technique. Since, priors were diffused, i.e., with very little information, posterior distribution is only due to the dataset. Because of that we did not explain the Bayesian ordered logistic regression in detail, as, it is expected that readers will familiarize themselves with the standard statistical tool elsewhere.  (b) Agreed. We have moved the sentence to the method. |
| 3. Table 1 does not show data about 95%CI. It is better to remove “[95%CI]” in Table 1. | Agreed and removed. It was our silly mistake. |
| 4. In Figure 1, what does each line represent? Is it travelling routes? Are there seven routes? And please clarify what Thorangla, Damodarkunda Korala, Ladar, Muktinath Tsarang, Hospital, Kathmandu, and Pokliara represents. | Each dashed line represents a travelling route. There are multiple routes in the area and travelers travelling on these different routes with different route characteristics come in to the hospital. Thoron La, etc. are either destinations or places in the route. We placed them there to give reader an idea about elevations of different trekking destinations/way points in the area. Hospital is the Mustang district hospital whose record were used in this study. It is at an elevation of 2700m.  We have updated the figure caption for clarification of issues raised in this comment. |
| 5. In Table 2, what does every data represent? (log odds?) What are “Cut point 1”, “Cut point 2”, “WAIC”, and “PSIS”? Could you please clarify these? | Yes, they represent log odds for four model specifications. We, now, have clarified about coefficients, WAIC and PSIS in the table’s footnote. Cut point 1 and cut point 2 are intercepts of ordered logistic regression (There are 2 cut points because our outcome has three levels). |
| 6. In the first of the Result, could you please describe how many candidates were included in this study. | We now have changed the result appropriately. |
| 7. In 3.1 Sociodemographic characteristics, what do Ｘ2 and df represent? Please clarify these words or remove them. | χ^2^, *df, and* *p* are result of the chi-squre test of variable with the severity of AMS. χ^2^ stands for chi-squre statistic and df is degree of freedom. Since, they are reporting of standard statistical technique commonly used in the literature, we don’t think appropriate readers will be confused by them. |
| 8. P-value of smoking history was <0.01 in 3.1 Sociodemographic characteristics, but 0.015 in SI Table1. Which one is correct? | It is typo. The SI table result is correct. We have changed appropriately. |
| 9. Please change “Km” to “km” in Table 2, Figure 1 and 2. | Agreed and changed appropriately. |
| 10. Please describe the limitations of this study in the Discussion. I think the content you mentioned in 2.7 Bias is a limitation. | We agree with this comment. In bias section, we were describing limitations of the study. Following the suggestion, we have removed the bias section from Methods and added one new limitation related paragraph at the end of the discussions. |
| **Reviewer 2 comments:** | **Response** |
| This manuscript reports the results of a retrospective study analyzing hospital admission for emergencies for the presence of altitude illnesses (AMS, HACE and HAPE) in a remote high-altitude region in Nepal. The authors are commended for their work which provides a welcome addition to the literature in this field. |  |
| Major  What is the reason that the authors did not include BMI in their analysis? Given they had full access to the hospital records I presume that stature and body mass were available. If they have collected those data I strongly suggest to include BMI (or a similar compound variable) in their analyses and report the results. | We did not had access to full hospital records. Rather, we had access to a specific form that was filled for AMS patients. The original purpose of the form that we used for the analysis was to standardize the diagnostic routine. The form did not collect height and weight of the patient. So, BMI could not be calculated.  Following your important comment, we have added few lines in the beginning of limitation related paragraph in the end of the discussion. |
| Minor  L2, perhaps better to write ‘entries for high-altitude sickness’  L24-25, I suggest: ‘Our study connects ascent rates with prevalence and severity of acute mountain sickness (AMS) among patients admitted to the emergency ward of the Mustang district hospital in Nepal.’  L29, in the abstract I suggest to omit the decimals of the percentages  L40, I suggest to add ‘can’ before ‘affect’  L43-44, I suggest: ‘to such a severe degree that they appear unable to acclimatize fully and must therefore descend.’  L46, ‘several weeks to months’  L51, ‘a rare but possibly fatal condition’  L56, as written the permanent residence is a bit confusing. I understand what the authors try to convey, but it is a bit unclear, please reformulate.  L59, use subscript in the molecule di-oxygen  L91, ‘the emergency ward of the Mustang’  L105, ‘The Mustang district hospital uses’  L106, ‘included’  L111, perhaps add that the data were anonymized.  L120, HACE without article.  L149, add anonymization  L167, ‘was lower’  L196, add ‘negatively’  L198, ‘while sex and’  L221, delete ‘would’  L226, ‘been a mixed bag’  L237, ‘at the Mustang district hospital’ | Thank you for patiently going through the manuscript. We sincerely appreciate it.  We have made almost all corrections bar one. On L105 comment, the hospital no longer uses the form that we used for analysis. So, we kept it as ‘The Mustang district hospital used’. |
| References, some missing references to estimations of altitude illness in that region, especially around the Thorong La | We have added new citations from specifically that region. Some of them were very pertinent, omitting them was our oversight. |
| Table 1, missing 95% CI, needs legend explaining what Smoking history, Alcohol intake and Medication history stand for | We initially had 95% CI in previous version. But we removed it to minimize the clutter. The heading was mistakenly left unedited. We have removed the 95%CI from column heading.  Agreed about legend. We have now updated the table footnote to explain about variables. |
| Table 2, spell out WAIC and PSIS in the legend | Agreed. We have put explanation on table footnote. |
| Figure 1, I believe it is Thorong La? | Agreed. We have changed it. It was a typo. |
