# Supplementary material for: Ascent rate and the Lake Louise scoring system: An analysis of one year of emergency ward entries for high-altitude sickness at the Mustang district hospital, Nepal
Source: PLoS One. 2022 Oct 27;17(10):e0276901. doi: 10.1371/journal.pone.0276901 (PMC9612449; doi:10.1371/journal.pone.0276901)
Supplement: Attachment — Submitted filename: Response to reviewers (minor revision round2).docx [file pone.0276901.s006.docx]

| **Editor's comments:** | **Response** |
| --- | --- |
| Please respond to the comment form the reviewer who suggests "minor revision". It seems that there are some errors in terms of a finger space, especially in the results area. For example, L155, %of etc. | Thank you for your kind suggestions. We have corrected as suggested. |
| **Reviewer 1 comments:** | **Response** |
| 1. Thank you for modifying Figure 1 capture. However, Figure 1 is still a little difficult to understand. For example, it is not clear if people pass through the Hospital when they are climbing or when they are descending (or both?). It is also unclear whether people pass through Ledar in all routes. How about using marks or colors such as ☆, ＊, ■, …etc. for Thorong La, Damodarkunda Korala, Ledar, Muktinath Tsarang, Hospital, Kathmandu, and Pokhara? I think it will be clear where to pass at which point in the figure. Moreover, the overlap between the line of routes and the word “Kathmandu and Pokhara” is not good and should be modified. | The aim of the figure 1 is to show ascent profile and roads types faced in the various traveling routes. On top of it, we added location names against altitude to give readers sense of the elevation of way-points.  Following your suggestions, we have changed the figure for:   1. Kathmandu and Pokhara no longer overlap the lines. 2. We added color rings for waypoints that are listed. But we did not used different symbols. We tested the figure, but found that using different shapes cluttered the image too much. Also, we have not marked hospital in the path. Rather we have added a clarification in the figure caption regarding whether people pass hospital while ascending or descending. |
| 2. Thank you for explanation of Table 2. You described “AMS severity” in the first row of Table 2. However, I think “logistic model” or “logistic model of AMS severity” is better and more understandable because (1), (2), (3), and (4) in the second row shows not AMS severity but logistic model. Please consider correcting if my interpretation is correct. | We have changed as suggested. |
| 3. In Table 2, I understood that Cut point 1 separated between None and Mild and that Cut point 2 separated between Mild and Moderate or Severe. How about describing like “Cut point 1 (None vs Mild, Moderate or Severe)” and “Cut point 2 (None or Mild vs Moderate or Severe )” if my interpretation is correct. | The intercept in ordered logistic model are in log-cumulative-odds scale and stand in for the cumulative probability of each outcome. The log-cumulative-odds of a response value $y_{i}$ is equal to or less than some possible outcome value $k$ is :  $log\frac{Pr\left( y_{i}\leq k \right)}{1-Pr\left( y_{i}\leq k \right)}=\alpha_{k}-\beta x_{i}$  Where $\alpha_{k}$ is intercept unique to each possible value $k$. But, by math we only require $n-1$ intercepts (The last intercept comes free). The problem is $\alpha_{k}$ impact only relative scaling (i.e., other $\alpha$ and has no impact on $\beta$ (subject of our interest). As a result, the magnitude and sign of the $\alpha$ depends on how the programmer implements the ordered logistic regression in software. Because of this, we cannot entirely rule out that if somebody runs this regression with same dataset with other software, they might get $\alpha$ with different magnitude or sign. The key being implementation detail.  An interesting exercise (in Bayesian realm) is to have different prior distributions with diverse means to sample $\alpha$ from. One will get same $\beta$ but completely different sets of $\alpha$ (We tested it). Since, there is no guideline on choosing prior of $\alpha$ and its posterior depends on the prior, ascribing any special meaning to it is difficult. Because of this, we don’t feel comfortable describing the cut points as suggested. Further, sign and magnitude of cut points in ordered logistic regression don’t offer natural meaning. |
| 4. You answered that the odds ratio is calculated as ratio of P(1day\|X)/ P(3day\|X) in Fiugre 2 and that you have explained textually about the odds ratio. However, I think Figure 2 doesn’t show that the odds ratios of ascent rate and smoking are 3.13 and 0.16, respectively, which are described in the Abstract. Does Figure 2 reveal the odds ratio of smoking? Moreover, the Figure 2 caption did not explain about odds ratios of ascent rate and smoking. The result described in the Abstract should be also written in the manuscript, Table, Figure, or Figure caption. Please reconsider. | Thank you for pointing out our oversight. The odds ratio in the abstract are not from the counterfactual plot, but from the regression table. We reported log-odds in the table but reported odds-ratio in the abstract. We have now added odds-ratio in the relevant section of the result. |
| **Reviewer 2 comments:** | **Response** |
| I thank the authors for addressing all of my comments and suggestions. I have no more additional points to make. | Thank you for your kind response. |
